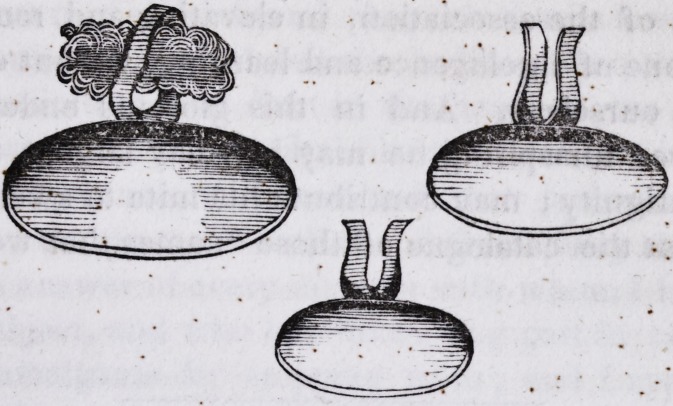# Observations on Artificial Obturators and Palates

**Published:** 1846-03

**Authors:** B. B. Brown

**Affiliations:** St. Louis, Mo.


### ARTICLE V.

*Observations on Artificial Obturators and Palates.* Read before the Mississippi Valley Association, at Cincinnati, Ohio, August 19th, 1845, by B. B. Brown, M. D., D. D. S., of St. Louis, Mo.

Gentlemen:

At your last annual meeting, I had the honor to be one, among the number of those appointed, to prepare a paper to be read before you, at the present convention.

Until very recently, I entertained the hope of being with you, at this meeting, when an essay, on another subject, would have been submitted for your consideration; but, circumstances beyond my control, have compelled me to forego my expectation in that respect; and, I now merely propose to make some observations on fissures of the palatal arch and their treatment, by means of *artificial obturators and palates.*

In the catalogue of calamities, to which human nature is subject, there is none which excites sympathy, in a higher degree, nor more frequently calls into requisition the highest order of professional ability, than the destruction of those parts composing the face and mouth, whereby the physiognomy is rendered hideous and articulation indistinct. Syphilitic diseases and the abuse of mercurial medicines, constitute the most prolific source of these misfortunes. The unhappy victim lives awhile, courting death to end his sufferings; and, if he survives, he exists merely a wreck of what he was, tedious to himself, and, not unfrequently, an object of mingled pity and disgust to his friends. The loss of bony structure is most generally confined to the superior and inferior maxillary, the spongy bones of the nose, and those of the palate.

Caries of bone is accompanied with a destruction of the soft surrounding substance; hence, when the palate bones are the seat of caries, a fissure in the palatal arch is the inevitable consequence, and which, not unfrequently, involves the loss of large portions of the alveolar ridge, together with the accompanying teeth.

The condition of a patient thus situated, is truly deplorable, and calls for the commiseration and sympathy of the dental philosopher and philanthropist. Unable to articulate, or enunciate a word distinctly, the unhappy individual is alarmed at the unnatural and incoherent sounds which he produces, and which too plainly indicate a fissure of the palatal arch. Deglutition is rendered laborious and painful, in consequence of fluids and solids constantly entering the fissure and traversing the naris.

From the period when the palate bones become diseased, a considerable length of time must necessarily elapse, before the carious bone is sufficiently detached, to admit of its removal; hence, two varieties of fissure are frequently presented, which I would denominate *interrupted* and *uninterrupted.*

1st. A fissure is *interrupted* when the palate bones remain *in situ*, not being sufficiently detached by the suppurative process, to admit of their removal. The diseased bone is generally elevated a few lines above the natural concavity of the palatal arch.

2d. A fissure is *uninterrupted*, when the communication between the palatal arch and naris is free from obstruction :—the carious bone having been removed by the slow process of suppuration, or by the skill of the surgeon.

It becomes the province, then, of the dental surgeon to remedy the loss, caused by accident or disease, of this important partition, and thus restore the unfortunate sufferer to a full and proper use of his functions. This is accomplished by artificial means, known to the profession as *artificial obturators* and palates.

By *artificial obturators*, I recognize appliances intended to restore the lost functions of the palatal arch, consequent upon an *uninterrupted* fissure in the hard palate, and which appliances are introduced into the fissure. These may be divided into simple and complicated. The *simple*, are those which have for their object, merely, the stoppage of the fissure, without extending beyond the edges of the aperture, and which are retained in position by a variety of appliances, such as elastic springs, bolts, wings, gum elastic, metallic drums, sponge, cork, gum mastic, wax, &c. &c. The *complicated*, are intended to perform the offices of the simple, with the addition of artificial teeth attached to them, by extending a plate to the point on the maxillary ridge, where they are to be supplied. In addition to the appliances usually attached to obturators, sometimes, also, arms or clasps are employed for the purpose of retaining them in their place.

Artificial palates are likewise divided into *simple* and *complicated*, *entire* and *partial.* By *artificial palates*, I recognise appliances, intended to restore the lost functions, consequent upon fissures in the hard palate, (produced by accident or disease,) by covering them over. The *simple form* is without teeth, while the *complicated* has them attached. The *entire*, is that form which covers the roof of the mouth ; the *partial*, covers some portions of the palatal arch, only, and is retained in position, by arms or clasps, which connect it for support, with a tooth, or teeth, on each side of the jaw.

A *simple artificial* palate may be *entire* or *partial*, depending wholly upon the condition, extent, and position of the fissure.

A *complicated artificial* palate should *always* be *entire*, as the teeth, which are thereto attached, are thus rendered much more permanent and useful ; and it also gives the whole operation, a substantiality, not to be attained by any other projection.

Cases may occur, where there are no teeth in the superior jaw; then, I would recommend an *entire complicated artificial* palate, (single plate,) to extend as far back upon the soft palate as the patient can tolerate with comfort. The dental circumference of the plate, should pass, as far as possible, over the gum, up to where the muscles of the lip and cheeks unite with the jaw, in order to be retained in its place, by atmospheric pressure.

A few hours use of this plate, cannot fail, fully to acquaint the patient with the principle of its construction, and almost to restore him the lost functions of his original palate. And, in my opinion, this is one of the best and most complete methods known to the profession, of successfully adapting an artificial denture to the superior jaw, which will supply, with ease and comfort to the wearer almost the whole properties of the natural structure. I have introduced a number on this plan, during the last fifteen years, and in every instance with the most satisfactory results.

I have not, however, had the good fortune to meet with a case of fissure, accompanied with the loss of the superior denture; but the principle, I believe, is correct; and I entertain no more doubt of its entire success, in practice, than I do of any thing, which I have not fully tested.

In adapting an artificial palate to an interrupted fissure, the soft surrounding structure, will, necessarily, be in an extremely irritated condition; hence, there is but one alternative left for the practitioner to pursue, viz. to apply an *entire* artificial palate. This is demanded in order to distribute the pressure equally over the whole roof the mouth, and, consequently, to relieve the irritable parts as much as possible. A loop may be soldered to the convex face of the plate, fitted to embrace a bit of sponge, with a view to arrest the progress of matter into the throat, while the plate is to be retained in its place by arms clasping a tooth or teeth, on each side. If a complicated artificial palate is required, it should, as before remarked, be *entire;* the edges of the plate adapted to the teeth by means of uprights soldered to it, and projecting from two to three lines above it. Every inequality, which the surface of the teeth may present, must be accurately fitted, and yet, not to *press* upon them; the edges of the convex dental circumference should be carefully rounded off, so that no sharp edges will rest upon the gum. The plate should slip into its place, with the utmost ease and freedom—and be retained there, by means of arms attached to one or more good teeth on each side of the jaw. And from some experience in this matter, I am inclined to select, for this dental attachment, a position, if practicable, always *posterior* to the first bicuspides, as ensuring, in general, more stability and greater ease to the patient than an anterior one can.

Too much care cannot be observed in procuring correct models of the parts to be supplied. The whole mouth and teeth should always be taken, so as to exhibit their entire relations in every respect. Of several, which the careful operator will not fail to obtain, the one approximating most nearly to the original, should be selected as the working model; for the plate should always present every inequality of the surface, and fit it closely when introduced. Metal casts are frequently injured by a single blow from a heavy hammer, and are therefore no longer fit for use; indeed, for almost all operations, two or three pairs should be used, and the plate frequently annealed, during the process of striking up.

I am satisfied, that correct principles have often been abandoned through carelessness, or the want of mechanical tact in making the necessary preparations; and I may here remark, that if violence be done to principles bearing upon any of the surgical or anatomical relations of the operation, all the mechanism and finish which labor can bestow, must result in consigning the work back to the *crucible*, or, if permitted to go abroad, it will remain a monument of the operator's stupidity.

I will now cite a short history of a case, from my case book, illustrating the plan of a *complicated*, *entire*, *artificial palate*, introduced with a view of restoring the lost functions, consequent upon an interrupted fissure in the palatal arch.

In the spring of 1841, I was consulted by Mr. W. (then a resident of our city) relative to supplying him with an artificial palate. On examination, his mouth presented the following appearance: a large interrupted fissure occupied the centre of the palatal arch, and extended posteriorly to the transverse palate suture—

the true palate bones were already destroyed—the maxillary palate bone yielded slightly by pressing upon them, at that point, attended at the same time with a considerable degree of pain. The surrounding soft parts were in a state of inflammation, caused by the contact of carious bone and irritation, consequent upon deglutition. In the anterior part of the mouth the left lateral incisor tooth was gone, and the front incisor, of the same side, so much changed from its natural position, by the disease of its appendages, as to render it useless. The maxillary palate plate was carious throughout its whole extent, and hence the loss of the front teeth was caused by their proximity to the *foramen incisivum*, where there was a loss of some bony structure. The patient's constitution had suffered materially, and his general health was feeble.

The condition of this individual was lamentable, not only in conseqeunce of the difficulty he constantly experienced in taking nourishment, but from the abundant discharge of offensive matter from the diseased bone.

The annexed drawings of the model of the mouth, will convey some idea of the loss sustained, and the true position of parts.

Fig. 1.

FIG. 2.

Fig. 1. Model of the parts necessary for constructing the artificial palate; the fissure being filled up, and the roof of the mouth restored to its original concavity. 1. Extent of the fissure traced. 2. 2. Interstices which received the arms or clasps.

Fig. 2. Anterior view of Fig. 1, exhibiting the part to be supplied with artificial teeth.

(The drawings correspond, in every particular, with the mouth of the patient.)

He informed me that he had sought relief from a number of members of the dental profession, in different sections of the country, but without success, except in a single instance, when a dentist in the south fitted him with an entire artificial palate, which had been attended with some relief. This was a rude, unfinished piece of dental mechanism, its greatest fault being a want of concavity, which did not admit a sufficient play to the tongue, and, therefore, the pressure which it exerted was confined to the alveolar ridge. The sharp edges of the plate excited almost constant inflammation and ulceration of the gum; still, the principle, as far as it went, was correct. The patient was wearing this temporary plate when he applied to me.

I undertook the management of his case, and removed the front incisor tooth, which I have already alluded to, and instituted local and general treatment, until I was satisfied that the disease was cured, which caused the destruction of the parts. As soon as the most urgent symptoms attending the case were controlled, I proceeded to model the mouth, with a view of supplying a *complicated, entire, artificial* palate. It will readily be perceived, that there was but one method whereby this operation might be accomplished correctly, viz. by distributing the pressure of the plate equally over as large a surface of healthy structure as possible, so as to relieve the parts over the diseased bone; and that, when undue pressure should be exerted upon it, its force would be expended upon the maxillary walls.

Accordingly, I constructed the palate, and adapted its dental circumference to each tooth, by means of uprights of plate, soldered on so as to fit their various inequalities. A separation had been made, by a former operator, between the second bicuspides and first molars of each side; there, I placed the arms, and constructed them to clasp the molars. In consequence of the arms of the former plate having clasped the bicuspides, I was subsequently induced to attach the clasps to them, as there could be no real objections, and, besides, the comfort of the patient was materially augmented. The clasps were as wide as the teeth to which they were attached. The artificial teeth were porcelain, with imitation gums. The plate was gold, 20 carats fine, and weighed 12 dwts.; it possessed the same concavity which was natural to the roof of the mouth. On the convex side a loop was soldered, to embrace a bit of sponge, so as to arrest the discharge of matter into the throat. It was introduced into its place in the mouth, on the 25th of May. All who witnessed its introduction, (several medical friends were present,) were much gratified with the result, and the wearer was unceasing in his expressions of gratitude.

The annexed drawings will convey a tolerable idea of the operation.

Fig. 1. Complicated, entire, artificial palate, view of convex face. 1. 1. 1. 1. Dental circumference. 2. 2. Arms or clasps. 3. Loop. 4. 4. Base upon which the artificial teeth rest.

Fig. 2. View of the concave face of Fig. 1. 1. 1. Artificial front and lateral incisor teeth, posterior view. 2. 2. Uprights connecting the teeth to the gum plate. 3. 3. 3. 3. Uprights soldered to the dental circumference, and projecting above the level of the plate two and a half lines. 4. 4. Corrugations of the gum brought up.

Fig. 3. Anterior view of the front and lateral incisor teeth in Fig. 2.

(The drawings correspond, in every particular, with the original, except that the convexity and concavity is not quite faithful, or well developed.)

Fig. 1.

Fig. 2.

Fig. 3.

During a period of more than two years that the patient remained in St. Louis, I had frequent opportunities of examining the operation; its success was complete in every particular, which proved the correctness of the principle upon which it was constructed.

However, I still continued to give him professional advice, relative to his general health, and the diseased bone, with a view to its removal, when the proper time should arrive. Accordingly, on the 17th of April, 1843, the patient consented to the operation, and I proceeded to perform it. The bone being too large to be drawn though the fissure entire, I was forced to cut it into pieces, with instruments constructed expressly for that purpose, and thus effected its removal. In the course of one month he was *well;* the suppuration had ceased; no fetor; a subsidence of the constitutional symptoms; a restoration to the society of his friends; and, to use his own words, "he felt that he was a new being."

The annexed drawings will convey a good idea of the diseased bone, and instruments made use of to cut it.

Fig. 1.

Fig. 1. 1. Anterior nasal spine. 2. Portion of right nasal process, superior maxillary. 3. Palatine process, left superior maxillary bone. 4. Anterior extremity of vomer.

Fig. 2.

Fig. 2. Inferior view of Fig. 1, with figures corresponding. A. A. Where the bone was divided to effect its removal. B. B. The same as A. A. C. Posterior edge of the maxillary palate plate, the *palate bones* being destroyed up to their suture. D. D. Middle palate suture.

(The drawings are about three lines smaller than the original.)

Fig. 1.

Fig. 1. Plugging Forceps. 1. Shank, with a fine cutting edge at its extremity. 2. Shank, with a groove at its extremity, to receive the cutting edge or knife of 1. 3. View of the groove in 2.

Fig. 2.

Fig. 2. Cutting Forceps, with fine sharp edges.

(The drawings correspond, in every particular, with the instruments.)

It was necessary to reduce the drawing about two-thirds, in order to get on page.—*Ed.*

Dr. Solyman Brown, of New York, saw Mr. W. a few months after the operation was introduced, and while the diseased bone was *in situ.* I was subsequently informed that it excited the highest admiration and commendation of that distinguished dental surgeon.

The question very naturally arises, which plan is to be preferred, *artificial obturators*, or palates? The answer will depend upon the position, condition, class and extent of the fissure; as a general rule, however, artificial palates are to be preferred to artificial obturators.

*Artificial palates* should exert equal pressure over an extent of surface which is capable of sustaining it without inconvenience. From the plate, merely acting as a covering to the fissure, I have invariably found, that the size of the aperture was diminished, (when properly adapted,) always realizing the most sanguine expectations of effecting what was intended. Artificial obturators by reason of filling up the fissure, and pressing upon its walls, invariably enlarge the opening, and serve to keep up a continual state of irritation in the surrounding substance, I, therefore, deem these objections sufficiently strong to cause the total abandonment of the plan.

Artificial palates may be constructed to cover various extents of surface, always depending upon circumstances. As a general rule, however, when the *os palati* and *maxillary palate plate* are destroyed, an entire artificial palate is demanded; so, likewise, when circumstances occur, making a complicated operation necessary, the palate should, generally, be entire also. The plan of atmospheric pressure and general pressure, exerted by the edges of an entire plate, upon the natural teeth are objectionable. The principle of atmospheric pressure is only applicable to artificial palates, when the teeth of the superior jaw are all wanting, and then the advantages of the principle should be obtained by a single plate.

When the teeth are involved in the loss of the palate bones, by disease, they are generally the front incisors, and those immediately over the cavity of the antrum; but gun-shot, and other wounds may injure every class. As the teeth are often exposed to injury from external violence, the practice of connecting an obturator to a complicated artificial palate, is liable not only to the objections already stated, but also to the important one, which arises from the strong possibility, that this very injury may produce a still greater one, by causing the artificial palate to be forced or *driven*, into the *fauces*, so that the projection, or drum, which fills the fissure, may rupture the *velum palati*.

These results are not impossible, and afford good reason for abandoning the use of an appendage, against which, so many objections may be urged. It possesses no advantage over a plate covering the aperture. Whereas, a plate is free from the objections to such an appendage.

The object of placing a bit of sponge in a loop on the convex face of an artificial palate, is intended merely to arrest suppurative matter from passing into the throat. This becomes necessary when the fissure is interrupted. The sponge should be much smaller than the fissure, so that when it is charged with matter it may not press its parieties. To promote personal comfort it should be frequently changed or cleansed.

The substances proper to be used in the construction of artificial palates are gold, palladium, and platina; the two former are to be preferred, on account of the elasticity which they acquire in the process of working.

The *uprights* which I have already described, are indispensable to an *entire* artificial palate; and if the directions which I have laid down are properly followed, they will be found to avert inflammation and ulceration of the gum. When, however, they are omitted, almost constant inflammation attends the whole line of its dental circumference, by reason of the gum coming in contact with the sharp edges of the plate.

Ambrose Paré claimed to be the author of artificial obturators or "stopples," as he called them, and gave to the world the first account of such appliances, with drawings, about three hundred years ago.

"Figure of the instruments which are named, stopples of the palate of the mouth."

The above is an exact representation of the drawings contained in the great work of Ambrose Paré, on the subject of obturators, together with a faithful translation of what he named them.

But since that remote period, very great and valuable improvements have been effected in the treatment of fissures; so much so, indeed, that it would almost seem impossible for the ablest and most talented member of our profession, to advance nearer to *perfection*; the *ne plus ultra* of scientific research. And as it regards the particular class of appliances, here briefly, and I regret to say, imperfectly treated, they appear after the maturest deliberation, scarcely susceptible of further improvement. Yet, so great is my faith in the vast power to be acquired by close observation, and experiments, based on scientific principles, that I believe even deficiencies of the *velum palati*, will at some future period, perhaps not far distant, be remedied or supplied by artificial means.

If we may throw a glance over the history of the last fifteen years, how many subjects for proud congratulation will offer themselves to the devoted and pure minded practitioner of dental surgery? Its scientific works now fill libraries, its literature is enlarged, its mechanism, for beauty and utility of design, is unsurpassed; and its profession embraces some of the most talented, benevolent, and well read men in our country, who would honor any profession, any calling, in the land; and whose lives, I trust without vanity, I may say, are solemnly dedicated with a self-sacrificing spirit, rarely excelled, to the benevolent labors of ameliorating human suffering, and of rendering the profession of their choice, one of high consideration and usefulness to the whole human family.

Brethren of the association, in elevating and rendering our profession one of intelligence and learning, we but ennoble and distinguish ourselves. And in this glorious endeavor, every one, however unaspiring he may be, may largely advance its honor and dignity; may contribute his mite to swell its history and augment the catalogue of those "names that were not born to die."

## Figures and Tables

**Fig. 1. f1:**
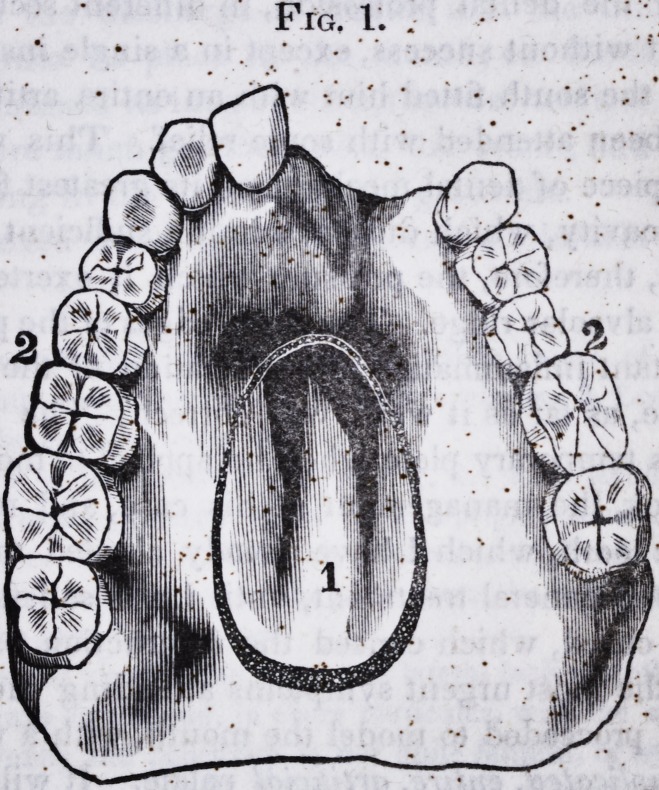


**Fig. 2. f2:**
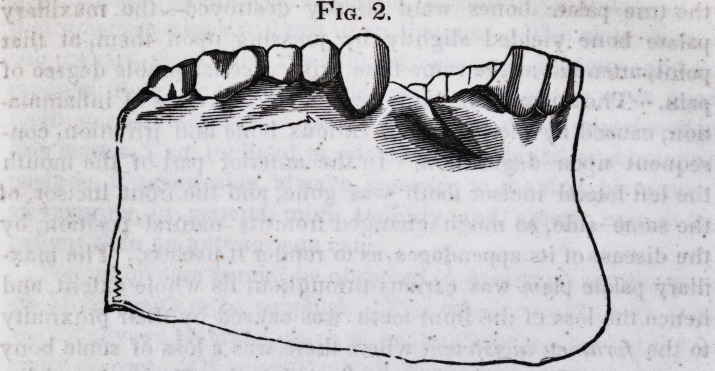


**Fig. 1. f3:**
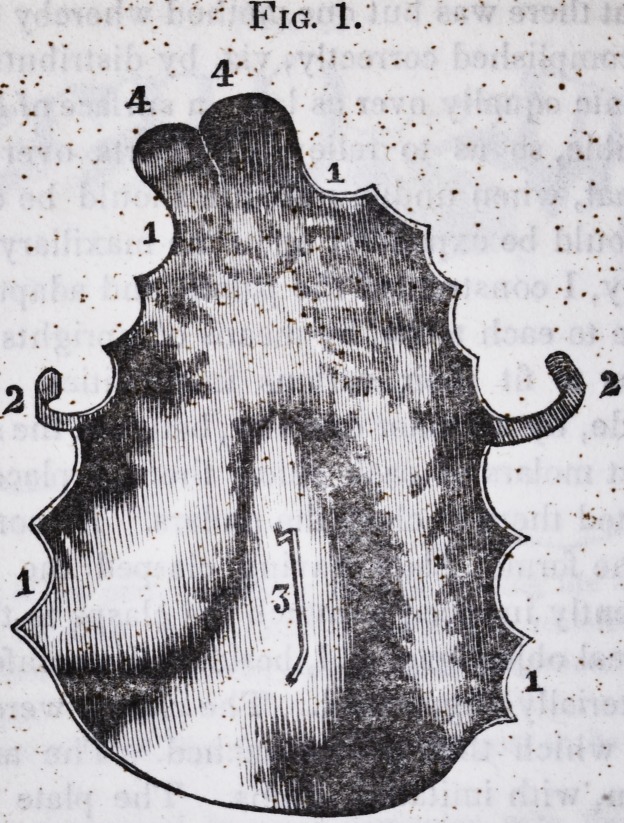


**Fig. 2. f4:**
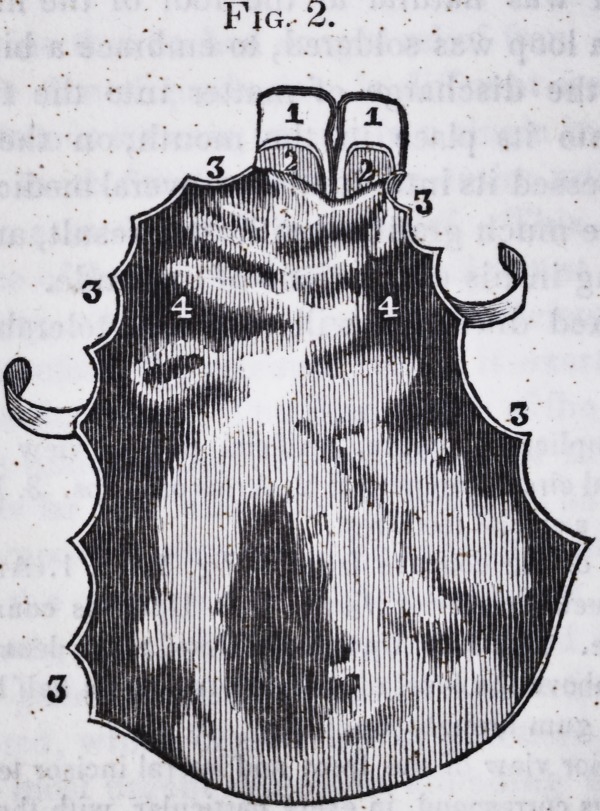


**Fig. 3. f5:**
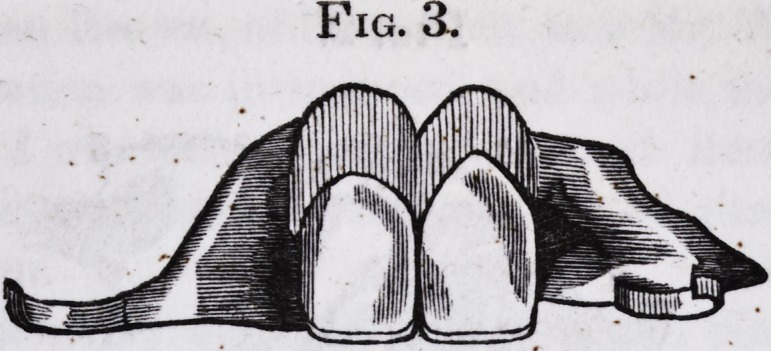


**Fig. 1. f6:**
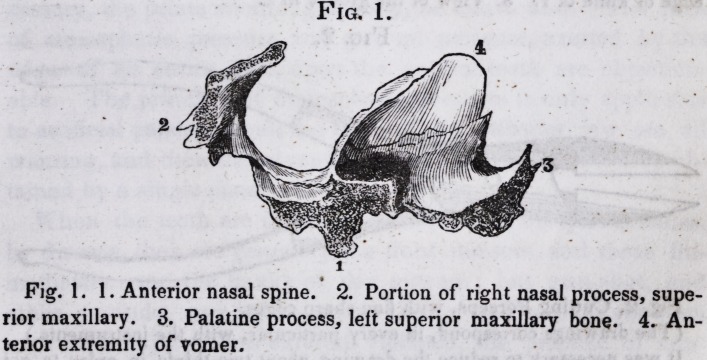


**Fig. 2. f7:**
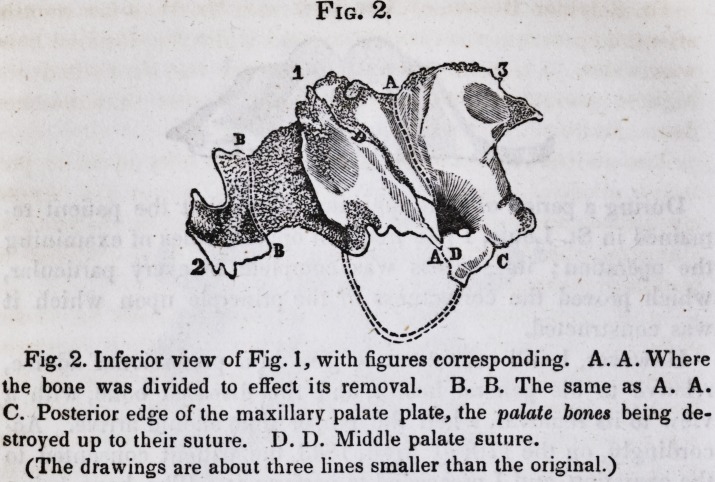


**Fig. 1. f8:**
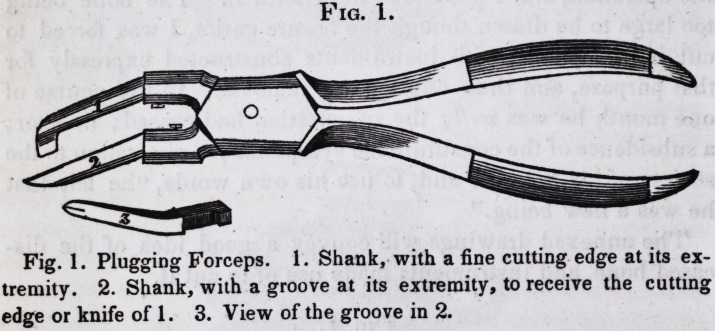


**Fig. 2. f9:**
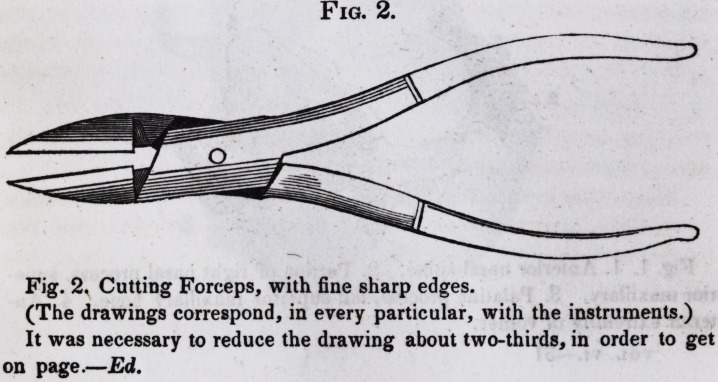


**Figure f10:**